# A New Quantitative Gait Analysis Method Based on Oscillatory Mechanical Energies Measured near Body Center of Mass

**DOI:** 10.3390/s22228656

**Published:** 2022-11-09

**Authors:** Derek Cheung, Jeff Cheung, Vicky Cheung, Li Jin

**Affiliations:** 1 Surge Motion Inc., Fremont, CA 94536, USA; 2 Biomechanics Research Laboratory, Department of Kinesiology, San José State University, San José, CA 95192, USA

**Keywords:** IMU, BCOM, gait analysis, oscillatory energy, energy partitioning

## Abstract

Human locomotion involves the modulation of whole-body mechanical energy, which can be approximated by the motion dynamics at the body’s center of mass (BCOM). This study introduces a new method to measure gait efficiency based on BCOM oscillatory kinetic energy patterns using a single inertia measurement unit (IMU). Forty-seven participants completed an overground walk test at a self-selected speed. The average oscillatory energy (OE) at BCOM during walking was derived from measured acceleration data. The total OE showed a positive correlation with forward-walking velocity. The ratio of total OE to constant forward kinetic energy for healthy adults varied from ~1–5%, which can be considered the percent of oscillatory energy required to maintain gait posture for a given forward-walking velocity. Mathematically, this ratio is proportional to the square of the periodic peak-to-peak displacement of BCOM. Individuals with gait impairments exhibited a higher percentage of oscillatory energy, typically >6%. This wearable IMU-based method has the potential to be an effective tool for the rapid, quantitative assessment of gait efficiency in clinical and rehabilitation settings.

## 1. Introduction

Human movement involves the motion of various limbs and segments. Traditional biomechanics research protocols analyze the kinematics and kinetics of body segments collected by a sophisticated camera-based motion capture system and force plates [1]. In translational movement analysis, such as walking and running, the whole body can be regarded as a system. The body’s center of mass (BCOM) can be regarded as the body mass concentrated at one virtual point and has been shown to be a good approximation for analyzing total body dynamics [2]. Extensive work has been carried out using ground reaction force (GRF) and 3D motion analysis of BCOM motion during gait to measure mechanical energy changes [3,4,5,6], gait pathologies [7,8], gait stability [9], gait efficiency in rehabilitation [10,11], and many other factors over the past few decades. In recent years, wearable inertial measurement units (IMUs) have been widely used to provide further insights into gait mechanics [1,2,12,13,14,15,16,17,18,19,20,21,22,23,24]. Although force plates and 3D optical systems are considered the gold standard for obtaining gait measurements, numerous studies in recent years have investigated the accuracy and validity of IMUs in estimating gait parameters. A variety of approaches and methodologies have been explored and can be summarized in three main categories: model-based [25,26], machine learning [27,28], and double integration [29,30]. Leveraging the advantages of IMUs and continued improvements in hardware and signal processing techniques, this study introduces a novel concept and a related algorithm to characterize gait energy efficiency and spatial energy balance using a single IMU. 

Previous studies have reported that the placement of an IMU sensor around the fifth lumbar vertebra could serve as a reliable approach for measuring BCOM kinematics during walking [1,12]. Based on Newton’s Law, the measured acceleration data from the IMU is used to derive various mechanical properties of BCOM motion. The results are consistent with measurements from 3D motion capture systems and force plates [2]. Forces acting on BCOM are translational only and can be decomposed into three orthogonal anatomical directions for further analysis: medio-lateral (ML), vertical (VT) and anterior-posterior (AP). Analyzing BCOM motion in each of these directions provides valuable information on the spatial energy balance.

In this research, we investigated BCOM oscillatory motion during walking and introduce the concept of oscillatory energy (OE) as a key parameter for the characterization of gait mechanical energy efficiency and spatial balance. 

During walking, in addition to average forward locomotion, the BCOM moves in a periodic oscillatory pattern in the ML, VT and AP directions [13,14]. In the ML direction, the oscillatory movement is a result of the BCOM, alternately shifting towards each landing foot during a stride. The zero-mean (mean value set to zero) for displacement falls on the centerline of the walking direction. In the VT direction, the up-and-down oscillatory displacement is a result of the modified inverted pendulum motion of the lower limbs and the need to create ground clearance. The zero-mean is the average height of the BCOM between the maximum and minimum position. The oscillatory motion in the AP direction is caused by the deceleration at heel strike and the re-acceleration at the push off and swing phase of each gait cycle. In the velocity domain, this oscillatory motion is an incremental modulation of the forward velocity superimposed over the constant-average forward velocity. At the steady state, the mean value of the oscillatory displacement is zero, which can be envisioned as the trajectory of the BCOM in the AP direction while walking on a treadmill moving at the same average forward velocity. None of these oscillatory motions directly contribute to the net forward locomotion, but are critical for maintaining proper gait form to enable a body’s efficient and stable forward motion. 

The average kinetic energy associated with the oscillatory motion, or OE, is directly derived from the measured acceleration, which contains fundamental information on the gait energy efficiency and spatial energy balance.

There are many published papers on gait analysis using wearable IMUs [1,15,16,17,18,19,20,21,22,23,24]. These studies focus mainly on using basic parameters such as acceleration, velocity, displacement, and spatial-temporal orientation. To our knowledge, no studies have examined the use of time-averaged kinetic energy from the oscillatory motion for gait analysis, which provides a powerful new perspective.

## 2. Materials and Methods

### 2.1. Participants and Protocol

A total of 47 older adult participants (68.2 ± 8.3 years, 28 females and 19 males) were recruited in this study. Of these, 38 participants reported themselves as being in excellent health with active lifestyles, while 9 of the participants reported some underlying health issue such as heart congestion, long-COVID, osteoarthritis, or back pain. Each subject signed an informed written consent form before the test. They were instructed to walk at a comfortable, self-selected speed along a 10-m walkway with a cone placed at the 10 m mark. Each subject walked around the cone and continued back to the starting line without stopping. We also conducted a separate test on a single healthy subject (male, age 72, height 1.67 m, body mass 62 kg). The individual walked with his normal gait on a treadmill at 4 different comfortable speeds between 0.89–1.57 m/s. The test was repeated three times for each speed, and the average OE of the 4 tests was calculated at each speed. In addition to the normal walk, the single subject also walked on the treadmill with several self-induced irregular gaits, including a wide stance, bouncy, and shuffling walk. The purpose was to qualitatively check the sensitivity of the measured OE to intentional gait variations. 

### 2.2. Data Collection

An Aspire Motion device equipped with a Bosch Sensortec IMU (Surge Motion Inc., Fremont, CA, USA) was used to measure the BCOM motion of each subject. 

The device was secured on each individual near the L5 with an elastic band that held it firmly in place. All results were derived from Nine Degrees of Freedom (NDoF) sensor-fused data at a sampling rate of 100 Hz. Subjects were asked to stand in a neutral pose for three seconds prior to walking. A rotation matrix was constructed during the neutral pose and applied to the raw gait data to calibrate for any sensor tilt with respect to the earth’s frame of reference. The data was split into two segments for data processing. The first two and last two steps close to the beginning and the end of the 10 m were excluded so that only steady state steps were used. Data processing was performed in Python 3.7 using the Math and SciPy python libraries. As the focus of this study was on the oscillatory movements around the BCOM, all frequencies below 0.3 Hz were filtered out to minimize IMU drift errors while preserving the measured gait data fidelity. The 0.3 Hz cut-on was significantly below the lowest frequency of the ML signal. In some cases, a high cut-off frequency (15 Hz) was applied to minimize high-frequency jitter noise.

In order to validate the accuracy of the device, repeated tests were conducted on 9 different individuals using Noraxon’s myoMOTION IMU (Noraxon USA Inc., Scottsdale, AZ, USA) mounted above the device. Values were in high agreement (r = 0.99). Intraclass correlation coefficient (ICC_2,1_—two-way random single measures) analysis were used to assess the validity of the derived acceleration, velocity, and displacement parameters. All ICC ranges were in excellent agreement (ICC > 0.92).

### 2.3. Data Analysis

When walking, the kinetic energy of the moving body can be calculated based on the acceleration measured at the BCOM. The acceleration is converted to velocity through simple integration. The three-dimensional velocity vector is:

(1) $$v(t)=\left[\begin{array}{c} {\bar{v}}_{ap}+v_{ap}\left(t\right)\\ v_{ml}\left(t\right)\\ v_{vt}\left(t\right) \end{array}\right]$$

where the velocity in the anteroposterior direction is decomposed into a constant term ${\bar{v}}_{ap}$ plus an oscillatory component $v_{ap}\left(t\right)$ whose mean value is zero. For the medio-lateral and vertical directions, the averages of $v_{ml}\left(t\right)$ and $v_{vt}\left(t\right)$ are also zero. The average kinetic energy for a gait of duration *T* is calculated as follows:

(2) $${\bar{E}}_{k}=\;\frac{1}{2T}\;\int_{0}^{T}∥v\left(t\right){∥}^{2}dt$$

Since the square of the norm of a vector is the sum of the squares of its components, ${\bar{E}}_{k}$ is:

(3) $$\begin{array}{ll} {\bar{E}}_{k} & =\frac{1}{2T}\int_{0}^{T}{\left({\bar{v}}_{ap}+v_{ap}\left(t\right)\right)}^{2}+v_{ml}^{2}\left(t\right)+v_{vt}^{2}\left(t\right)\;dt\\ & =\frac{1}{2T}\int_{0}^{T}{\bar{v}}_{ap}^{2}+2{\bar{v}}_{ap}^{2}v_{ap}\left(t\right)+v_{ap}{\left(t\right)}^{2}+v_{ml}^{2}\left(t\right)+v_{vt}^{2}\left(t\right)\;dt \end{array}$$

The term $2{\bar{v}}_{ap}^{2}v_{ap}\left(t\right)$ vanishes because $v_{ap}\left(t\right)$ is by definition a zero-mean signal. Therefore:

(4) $$\begin{array}{ll} {\bar{E}}_{k} & =\;\frac{1}{2T}\;\int_{0}^{T}{\bar{v}}_{ap}^{2}+v_{ap}{\left(t\right)}^{2}+v_{ml}^{2}\left(t\right)+v_{vt}^{2}\left(t\right)\;dt\\ & =\frac{1}{2}{\bar{v}}_{ap}^{2}+\frac{1}{2}\int_{0}^{T}v_{ap}{\left(t\right)}^{2}+v_{ml}^{2}\left(t\right)+v_{vt}^{2}\left(t\right)\;dt \end{array}$$

The first term in Equation (4) is the time-invariant forward translational kinetic energy $KE_{0}$. The second term is the sum of the oscillatory energies in three directions, or the total oscillatory energy, TOE. The integration period can be averaged over multiple gait durations (n·T), where *n* is the number of gait periods measured. Equation (4) can be re-written as:

(5) $${\bar{E}}_{k}=\;KE_{0}+(\mathrm{APE}+\mathrm{MLE}+\mathrm{VTE})$$

where APE, MLE, and VTE are the time-averaged oscillatory energies in the anteroposterior, medio-lateral, and vertical directions. 

We define the ratio between the TOE and $KE_{0}$ as the Overhead Energy Percentage, OEP:

(6) $$\begin{array}{c} \mathrm{OEP}=\;\frac{1}{{\bar{v}}_{ap}^{2}}\int_{0}^{T}v_{ap}{\left(t\right)}^{2}+v_{ml}^{2}\left(t\right)+v_{vt}^{2}\left(t\right)\;dt\\ =\;\mathrm{TOE}/KE_{0} \end{array}$$

Thus, the OEP is the ratio of total oscillatory energy and the average forward kinetic energy, $KE_{0}$. It can be viewed as the percent of energy required to sustain the proper gait form to maintain a constant forward velocity. A low OEP value implies higher gait energy efficiency as less oscillatory energy is needed to maintain the required speed.

## 3. Results

**Figure 1:**
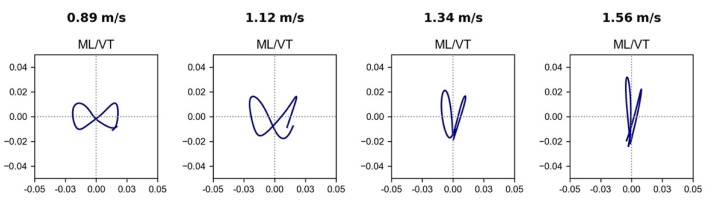
The ML/VT displacement trajectory of the BCOM during a stride. The vertical and horizontal axis indicate displacement in the VT and ML direction.

The focus of this research was on the oscillatory motion at the BCOM. To help visualize this movement during walking, we calculated its 3D displacement trajectory. The result was derived from time domain displacement equations based on measured acceleration data in each direction, which were then plotted as parametric equations using time as the independent parameter. The trajectories for a full stride in the ML/VT (frontal) plane at four different walking velocities are shown in Figure 1. Both the shape and the magnitude of the trajectory changed with walking speed. At normal walking speeds, the peak-to-peak oscillatory or periodical displacement of the BCOM in each direction was approximately 2.0 cm. The displacement in VT increased with speed but decreased in the ML direction.

**Figure 2:**
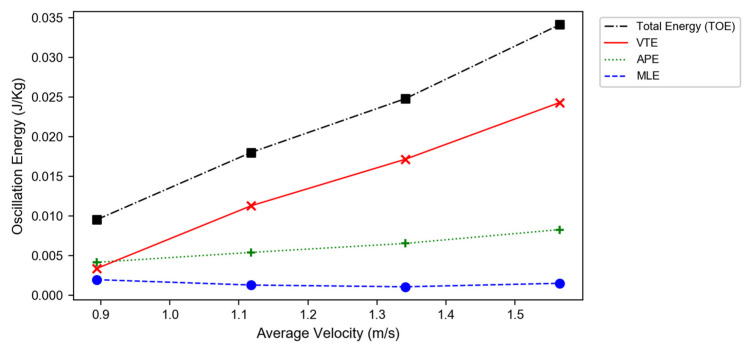
Oscillatory energy versus forward velocity at four walking speeds.

The OEs in the three directions (MLE, VTE, APE) were calculated based on Equation (4) and were plotted versus average walking velocity $V_{0}$ in Figure 2. The sum of the OEs, or the TOE, increased monotonically with walking speed—with most of the increase coming from the VTE. The changes in the APE and MLE were relatively modest. 

**Figure 3:**
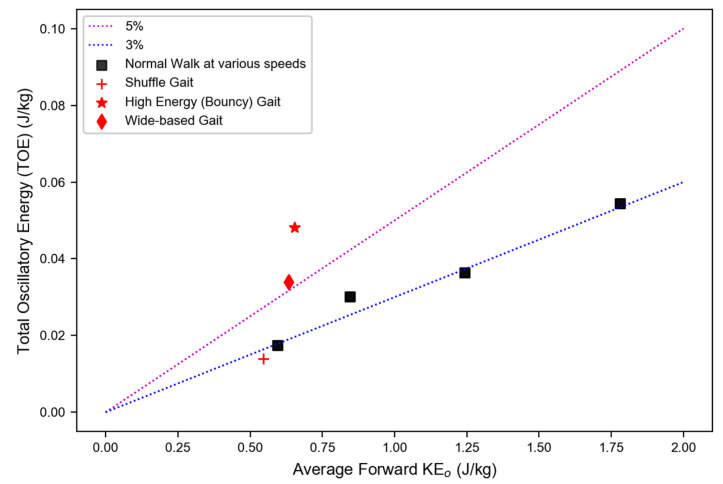
Total oscillatory energy (TOE) versus $KE_{0}$. The slope of the dotted lines is the OEP.

The dependence of the TOE on $KE_{0}$ is shown in Figure 3. For the single subject walking with normal gait, the TOE increased linearly with $KE_{0}$ over a broad range of comfortable walking speeds. The slope of the plot was the ratio defined by the OEP in Equation (6). In this case, the OEP was ~3%. Other data points from self-induced, simulated irregular gait were also included in the plot. Both the wide stance and bouncy gait deviated from normal gait behavior, showing a higher slope (OEP) compared to normal walking. 

**Figure 4:**
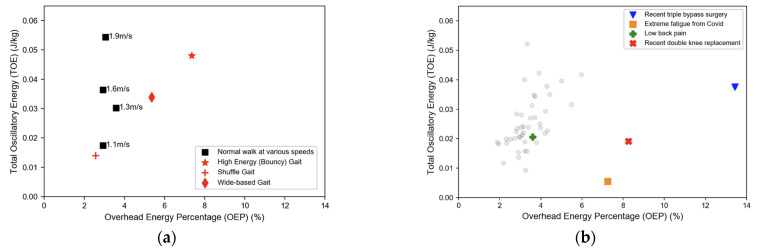
(**a**) TOE versus OEP based on the single individual from Figure 3. (**b**) TOE versus OEP of 47 subjects; 4 of the 9 participants who reported underlying health conditions are highlighted.

The TOE versus the OEP from the same individual are plotted in Figure 4a. The value of the OEP stayed within the range of 2.8–3.3%, even though the TOE variation was much broader. The OEP for shuffling gait was comparable to normal walking; however, the OEP for bouncy gait was >7% and wide stance gait was ~5.3%—both were significantly higher than normal gait at the same walking speed.

The TOE and OEP from the 47 subjects are plotted in Figure 4b. Each subject walked with a self-selected speed of 1.17 ± 0.23 m/s. The results of the healthy individuals were consistent with the single subject (mean OEP, 3.8%; Standard Deviation, 1.8%; range 1.9–5%). Individuals with a high OEP (>5%) all had known underlying health issues.

**Figure 5:**
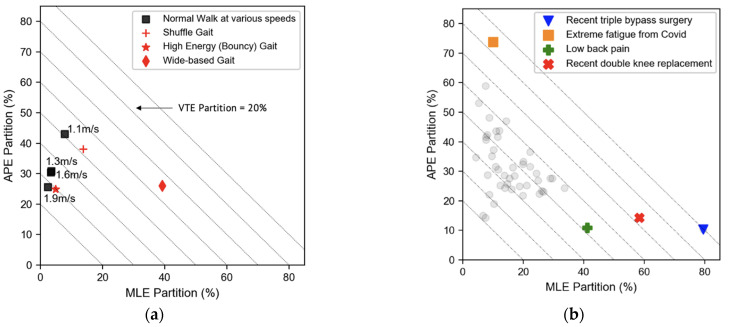
(**a**) Energy Partitioning graph of the single subject. (**b**) The EP graph of 47 subjects; 4 of the 9 participants who reported underlying health conditions are highlighted.

The partitioning of the OE in different directions provides additional information on the spatial balance of gait energy. The total OE portrayed in Figure 2 was replotted in a unique Energy Partition (EP) graph to depict the percentage of OE distribution in each direction at different walking speeds in Figure 5a. The *x*- and *y*-axis represent the percentage of OE distribution in the ML and AP directions, respectively (MLE% and APE%). The dotted diagonal lines are iso-VTE% contours and each line represents a constant value of VTE%. As the sum of the distribution percentage is always unity, any data point on the EP graph uniquely represents the percentage of OE in each direction. The EP graph enables the effective visualization of the OE spatial distribution. For the single subject at a walking speed of 1.3 m/s, approximately 30% of the total OE was in the AP direction, 66% in VT, and only 4% in ML. As walking speed increased, the VTE% increased continuously, reaching 72% at 1.9 m/s. Simultaneously, both MLE% and APE% decreased. For the simulated irregular gait, wide stance had a high MLE% of ~40%. The bouncy gait had an exceptionally high VTE% of 70%. For shuffling gait, the energy distribution was comparable to normal walking.

The EP graph of the 47 participants is shown in Figure 5b. The most striking result was that three out of the four outliers had the lowest VTE% at 10–28% and the highest OEP. All the highlighted individuals were from the group of nine participants who had reported health conditions. The participant who had bypass surgery and suffers from various health issues had the lowest VTE% and highest MLE% (VTE = 10%, MLE = 80%), with a concurrent high TOE (Figure 4b). The individual with a double knee replacement, with MLE% = 60%, also mentioned having right-ankle pain. The individual suffering from long-COVID fatigue had a significantly low VTE% (16%). Lastly, the individual in the normal OEP range, but who had a high MLE% (41%), complained of lower-back pain. The significance of this data is discussed in the next section.

## 4. Discussion

The core of this research was to analyze the oscillatory energy at the BCOM for gait analysis. We presented the in-depth behavior of OE using data from a single subject walking at four different speeds, which were then compared to the data from 47 subjects. 

To help visualize the BCOM oscillatory motion, the displacement trajectory of the BCOM in the ML/VT plane is shown in Figure 1. The bow-tie pattern was the result of the period for a ML cycle being twice that of the VT cycle [2]. The measured/derived trajectory was unique for each individual. In this particular case, the displacement in the VT and ML directions were dependent on walking speed. The peak-to-peak VT displacement increased from approximately 2 cm to 4 cm with increased speed but decreased in the ML direction. This observation was consistent with other studies [31,32].

In steady-state walking, the central nervous system purposefully maintains its forward locomotion at an average constant velocity $V_{0}$ with associated kinetic energy $KE_{0}$. For any individual, the forward translational motion is supported by a gait form, which is uniquely characterized by a periodic, oscillatory motion at the BCOM. The kinetic energy for this motion, OE, was calculated using Equation (4) for each subject. Note that the OE was the time-averaged kinetic energy over multiple (>10) steps. As such, its value is less susceptible to temporal transient errors or step-to-step variations and is, therefore, more accurate and repeatable.

The sum of the OEs in each direction increased with walking speed (Figure 2); however, the rate of increase differed in each direction. To understand this behavior, we need to examine the interactions between walking speed, step time (*T*), average oscillatory velocity in each direction, and peak-to-peak displacement (*d*). The approximate average oscillatory velocity for the BCOM displacement was ~2$\cdot d_{p-p}$/*T*. The factor of two takes into consideration the round trip. Thus, the oscillatory velocity depends on the ratio of total displacement to step time—both of which are dependent on walking speed. At faster walking speeds, the step time is shortened by the same amount in all directions. However, the magnitude of the peak-to-peak displacement in each direction—which is gait specific—has a more complex behavior [33]. For the single subject in Figure 1, his VT displacement increased with walking speed, which further amplified the vertical velocity beyond the scaled increase due to the shortened *T*; on the other hand, ML displacement decreased, which offset the effects of scaling. As a result, the oscillatory velocity and the related OE in the VT and ML directions scaled differently with walking speed. 

The dependence of the TOE on $KE_{0}$ is plotted in Figure 3. For the single subject, the ratio TOE/$KE_{0}$, or the OEP, was ~3% over a wide range of normal walking speeds. In other words, about 97% of the total average kinetic energy of the body was in forward locomotion and only 3% in oscillatory movement. In the Appendix A, we show that the OEP is related to the magnitude of the physical displacement of the BCOM. A value of 3% for the OEP corresponds to approximately 4 cm of BCOM movement in each of the three directions; the total displacement increases to approximately 6 cm at 6% OEP.

Another basic question is whether OEP is sensitive to gait variations. In Figure 4a, we included data from simulated irregular gaits. The OEP for shuffling gait was comparable to normal gait, but for wide stance gait the OEP was 5.3% and for bouncy gait it was 7.3%—both were significantly higher than normal gait at the same walking speed. Thus, qualitatively, the OEP was sensitive to gait variations, which is not surprising as it depends on total BCOM displacement. 

The OEP from 47 adults is shown in Figure 4b. The self-selected walking speed results were generally consistent with those of the single subject. For healthy participants, the OEP varied from ~2–5%. Some of the participants with underlying health conditions showed a higher OEP, ~6–13.4%. Deviations from normal gait, whether intentional or unintentional, led to a higher OEP. This demonstrates the potential for using OEP as a quantitative metric for gait efficiency assessment.

The spatial distribution of OE for the single subject at different walking speeds is shown in Figure 5a. The percentage of VTE increased from 50% at a walking speed of 1.3 m/s to ~70% at 1.9 m/s. At the same time, MLE% and APE% decreased from 8% to 4% and 44% to 26%, respectively. For the simulated irregular gait, wide-stance walking had a very high MLE% of ~40%; bouncy gait had a very high VTE% of ~70%. Based on the analysis from the Appendix A, the percent of OE in each direction is proportional to the square of the BCOM displacement in that particular direction, as a fraction of the total displacement. 

The EP graph for the 47 subjects is shown in Figure 5b. The distribution of the healthy participants are grouped in a region bounded by VTE% > 40%, MLE% < 35% and APE% < 50%; there is probably a fundamental biomechanical reason for requiring such an energy distribution to achieve optimal metabolic costs within the gait constraint. More systematic research is needed to link these mechanical results with kinesiological mechanisms [8,34]. Figure 5b also shows that three of the participants with known underlying health issues all shared a low VTE% (<28%), which was either due to slow walking or low BCOM vertical lift [8,32,33,34]. Two of the three had a low TOE (low energy level) with a concurrently high OEP (low efficiency). Healthy participants consistently exhibited higher VTE%, low MLE%, and modest-to-low APE%. Spatial energy balance information complements the use of OEP as a key metric for gait analysis. 

## 5. Conclusions

In this paper, we introduced a novel concept and a related methodology for assessing mechanical energy efficiency of gait based on measuring the oscillatory motion at the BCOM. The measurement is simple to perform and uses only one wearable IMU. Furthermore, the value of the core parameter, OE, is time-averaged over multiple steps; thus, the accuracy and repeatability are consistent. The key metrics used in the analysis—the overhead energy percentage (OEP) and energy partitioning (EP)—are both independent of body mass and are not sensitive to normal walking speed. Mathematically, these metrics are shown to be linked with the magnitude of oscillatory physical displacement of the BCOM during walking. All these attributes make this wearable IMU-based method potentially effective for quick screening of gait deficits for clinical evaluation [35] and for monitoring the efficacy of rehabilitation programs [19]. 

Most of the reported data are observational in nature. More systematic research is required to refine this technology and to establish a large and reliable database for specific applications. The linkage of quantitative BCOM mechanical properties to kinesiological and physiological research will help in fully realizing the potential of this novel methodology for a broad range of gait analysis applications and beyond.

## Data Availability

The data that support the findings of this study are available upon request from Vicky Cheung (V.C., vicky.cheung@surgemotion.bio).

## Appendix A. Oscillatory Energy and Periodical BCOM Displacement

The physical significance of OEP can be viewed from a different perspective from the following analysis.

The average velocity of oscillatory motion at BCOM in each direction can be approximated as:

(A1) $$V_{ave}\approx 2\cdot d_{p-p}/T$$

where $d_{p-p}$ is the peak-to-peak displacement of BCOM in the particular direction and *T* is the step time. From Equation (A1), *TOE* at a fixed $V_{0}$ can be approximated as:

(A2) $$\begin{array}{ll} \mathrm{TOE} & \approx \frac{1}{2}\cdot \sum V_{ave}^{2}=\frac{1}{2}\cdot 4\cdot \;(d_{vt}^{2}+(\frac{1}{2}\cdot d_{ml}{)}^{2}+d_{ap}^{2})/T^{2}\\ & =(\frac{1}{2}\cdot V_{0}^{2})\cdot 4\cdot [d_{vt}^{2}+(\frac{1}{2}\cdot d_{ml}{)}^{2}+d_{ap}^{2}]/\lambda^{2} \end{array}$$

All *d*’s are peak-to-peak displacement of the BCOM during each step. Only half of ML peak displacement is included since its period is twice the step time. Furthermore, step time *T* is substituted by its equivalent λ/$V_{0}$, where λ is the step length at walking speed $V_{0}$. Notice that the first term in Equation (A2) is $\frac{1}{2}\cdot V_{0}^{2}$, which is just $KE_{0}$. So mathematically we can re-state Equation (A2) as:

(A3) $$\mathrm{OEP}=\mathrm{TOE}/KE_{0}≅4\cdot \;[d_{vt}^{2}+(\frac{1}{2}\cdot d_{ml}{)}^{2}+d_{ap}^{2}]/\lambda^{2}$$

So OEP is proportional to the square of total BCOM displacement normalized to step length. Using Equation (A3) and assuming peak-to-peak displacement in all three directions to be approximately 4 cm and step-length of 70 cm, then OEP is ~2.9%. When displacement is increased to 6.0 cm, OEP jumps to 6.6%. These estimations are consistent with the more rigorous and accurate calculation for OEP based on Equations (4) and (5).

Based on Equation (A2), the percent of OE in each direction (APE%, VTE% and MLE%) can be viewed as the ratio of the square of its peak-to-peak displacement to the square of the total BCOM displacement. For example, VTE% is equal to $d_{vt}^{2}$/($d_{vt}^{2}$ + ${\left(\frac{1}{2}\cdot d_{ml}\right)}^{2}$ + $d_{ap}^{2}$). A high value of VTE% implies that proportionally the BCOM move more in the vertical direction relative to ML and AP.

This new perspective links OEP to total oscillatory BCOM displacement which can help to provide a more physical understanding of its significance.
